# Evaluating the Accuracy of Methods for Detecting Correlated Rates of Molecular and Morphological Evolution

**DOI:** 10.1093/sysbio/syad055

**Published:** 2023-09-11

**Authors:** Yasmin Asar, Hervé Sauquet, Simon Y W Ho

**Affiliations:** School of Life and Environmental Sciences, University of Sydney, Sydney, NSW 2006, Australia; National Herbarium of New South Wales (NSW), Royal Botanic Gardens and Domain Trust, Sydney, NSW 2000, Australia; Evolution and Ecology Research Centre, School of Biological, Earth and Environmental Sciences, University of New South Wales, Sydney, NSW 2052, Australia; School of Life and Environmental Sciences, University of Sydney, Sydney, NSW 2006, Australia

**Keywords:** Evolutionary rates, morphological evolution, macroevolution, molecular clock, simulation, flowering plants

## Abstract

Determining the link between genomic and phenotypic change is a fundamental goal in evolutionary biology. Insights into this link can be gained by using a phylogenetic approach to test for correlations between rates of molecular and morphological evolution. However, there has been persistent uncertainty about the relationship between these rates, partly because conflicting results have been obtained using various methods that have not been examined in detail. We carried out a simulation study to evaluate the performance of 5 statistical methods for detecting correlated rates of evolution. Our simulations explored the evolution of molecular sequences and morphological characters under a range of conditions. Of the methods tested, Bayesian relaxed-clock estimation of branch rates was able to detect correlated rates of evolution correctly in the largest number of cases. This was followed by correlations of root-to-tip distances, Bayesian model selection, independent sister-pairs contrasts, and likelihood-based model selection. As expected, the power to detect correlated rates increased with the amount of data, both in terms of tree size and number of morphological characters. Likewise, greater among-lineage rate variation in the data led to improved performance of all 5 methods, particularly for Bayesian relaxed-clock analysis when the rate model was mismatched. We then applied these methods to a data set from flowering plants and did not find evidence of a correlation in evolutionary rates between genomic data and morphological characters. The results of our study have practical implications for phylogenetic analyses of combined molecular and morphological data sets, and highlight the conditions under which the links between genomic and phenotypic rates of evolution can be evaluated quantitatively.

Evolution has generated the great diversity of phenotypic forms across the Tree of Life. However, the genetic mechanisms that underlie changes in phenotype remain incompletely understood (Orr 2001). It is often assumed that there is only a weak link between molecular and morphological change (Stanley 1975), given that the sheer number of mutations accumulating in genomes is likely to include just a small proportion that cause phenotypic changes (Gillespie 1991; Bromham et al. 2002). Furthermore, the proposal of the neutral theory of molecular evolution (Kimura 1968), which describes a large fraction of mutations as having negligible impact on an organism’s fitness, has bolstered the idea that molecular and morphological evolution are broadly decoupled. This possibility has been supported by the results of recent studies of empirical data (Lee et al. 2013; Halliday et al. 2019; Simões et al. 2020b). Although genetic drift is believed to be a substantial driver of molecular evolution (Kimura 1968; Ohta 1992), morphological characters, given their importance to an organism’s survival, are often assumed to be under strong selection (Lee and Palci 2015; Ho et al. 2017; Manceau et al. 2020).

Explicit tests of the link between genetic change and phenotypic change have largely focused on model species, mapping the effect of single genes or small sets of genes (Ashton et al. 2017; Kemble et al. 2019). Recent advances in genomic sequencing have propelled studies of quantitative trait loci, where genomic regions that account for phenotypic trait variation within a species can be identified. However, these studies are time-consuming, costly, and often lack statistical power (Ashton et al. 2017). A more efficient approach that can accommodate hundreds of taxa is to assess the link between rates of molecular and morphological evolution using phylogenetic comparative methods. If there is a correlation between rates of molecular and morphological evolution, then phenotypic traits might be predominantly governed by drift rather than adaptive processes (Halliday et al. 2019). Alternatively, a lineage with a high rate of molecular evolution might experience a high rate of morphological evolution simply because it has been exposed to larger numbers of phenotype-altering mutations. Such correlations in rates can be detected using phylogenetic methods, regardless of whether they are driven by evolutionary change along branches or by bursts of change at speciation events.

Correlations between molecular and morphological rates have been demonstrated in the evolution of mandibles in rodents (Renaud et al. 2007) and crania in primates (Ackermann and Cheverud 2004). The implications of such rate correlations depend on whether they are seen in protein-coding genes or regulatory regions of the genome. For example, a correlation in evolutionary rates between regulatory regions and morphological traits would suggest that those traits are under selection (Carroll 2008; Simões et al. 2020b). However, apparent correlations between molecular and morphological evolutionary rates might be due to both being driven by a third factor. For example, species with small populations might experience rapid evolutionary change because of the heightened impacts of drift on both genomic mutations and morphological traits (Combosch et al. 2017).

To date, there have been few explicit tests of the link between molecular and morphological evolutionary rates (Seligmann 2010; Parins-Fukuchi et al. 2021). Instead, indirect tests have been carried out on “living fossils,” or taxa with presumed morphological conservatism across long timescales and high evolutionary distinctiveness (Lidgard and Love 2018; Turner 2019). For instance, the tuatara (*Sphenodon punctatus*) diverged from squamates 260–245 million years ago (Ma) and is the only extant member of the order Rhynchocephalia (Simões and Pyron 2021). Despite the inference that the long lineage leading to the tuatara has experienced little morphological evolution (Herrera-Flores et al. 2017; Simões et al. 2022b), the mitochondrial control region of the tuatara has been estimated to evolve at a remarkably high rate (Hay et al. 2008; Subramanian et al. 2009). Earlier work on morphologically conserved horseshoe crabs showed only modest reductions in the rate of mitochondrial evolution compared with scorpions and brine shrimp (Avise et al. 1994). Furthermore, analyses of *Ginkgo biloba*, the sole surviving species in its order, showed enrichment in duplicated genes and expansion of gene families, bolstering complex chemical defence mechanisms against herbivory (Guan et al. 2016; Šmarda et al. 2016). These results suggest a decoupling of the rates of molecular and morphological evolution. In contrast, however, recent transcriptomic analyses of “living fossils” have shown reduced rates of molecular evolution across hundreds of protein-coding genes in *Nautilus* (Combosch et al. 2017; Huang et al. 2022), African coelacanth (Amemiya et al. 2013), tuatara (Gemmell et al. 2020), and long-lived sacred lotus (Ming et al. 2013).

Phylogenetic studies of the relationship between molecular and morphological evolutionary rates have produced a mixture of results. An analysis of 13 vertebrate data sets yielded no evidence for an association between rates of molecular and morphological evolution (Bromham et al. 2002), contradicting the results of an earlier study that detected such a correlation in 7 of the 8 diverse data sets analyzed (Omland 1997). Although most studies have tested evolutionary rate correlations in animals, analyses of small data sets from angiosperms (flowering plants) have found weak but positive correlations between rates of molecular and phenotypic evolution (Omland 1997; Barraclough and Savolainen 2001; Davies and Savolainen 2006). These positive correlations have been found across many angiosperm taxa, including: *Sedum* (family Crassulaceae), *Krigia* (family Asteraceae), birch (family Betuleacae), spindle trees (family Celastraceae), Hypoxidaceae, walnuts (family Juglandaceae), *Protea* (family Proteaceae), buckthorns (family Rhamnaceae), and monocots.

The persistent uncertainty about the relationship between molecular and morphological rates is compounded by the lack of a detailed investigation of the conditions under which a correlation can be detected (Simpson 1944; Seligmann 2010; Parins-Fukuchi et al. 2021). Without the use of post hoc power analyses (e.g., Davies and Savolainen 2006), it is unclear whether insufficient statistical power has precluded detection of correlated rates or if there is a genuine lack of an association. This is especially pertinent given that previous broad-scale studies examined small sets of genetic markers, commonly “housekeeping” genes (Omland 1997; Barraclough and Savolainen 2001; Davies and Savolainen 2006). These genes might not be well-suited for comparison with morphological rates, since they are under functional constraints and are unlikely to be representative of genome-wide patterns (Bromham et al. 2002; Davidson and Erwin 2006; Carroll 2008; Wagner 2014). Furthermore, morphological data sets often have features that hinder the efficacy of statistical analyses, such as more extreme and more complex patterns of rate variation, and small numbers of characters compared with molecular data sets (Uyeda et al. 2016; Pyron 2017; Stull et al. 2021). Thus, it remains unclear whether the mixed results from previous studies have been due to the use of different data sets, insufficient statistical power, or the use of varied methods to test for rate correlations.

Here we aim to uncover the conditions under which correlations between molecular and morphological rates of evolution can be detected. We present a comprehensive simulation study based on parameters from angiosperms, which lends reality to our estimates. We evaluate 5 approaches for detecting correlated rates of evolution under these conditions, including root-to-tip distance correlations, phylogenetically independent sister-pairs contrasts (Felsenstein 1985; Bromham et al. 2002), likelihood-based model selection (Duchêne et al. 2020), correlations of Bayesian branch rates inferred using relaxed clocks (Lee et al. 2013), and Bayesian model selection (Kass and Raftery 1995; Baele et al. 2016). Using the insights provided by the simulation study, we test for evolutionary rate correlations between angiosperm floral characters and genomic DNA. Our analyses have implications for understanding the relationship between genotypic and phenotypic change and inform practical recommendations for detecting correlated evolutionary rates.

## Materials and Methods

### Simulations of Molecular and Morphological Evolution

#### Phylogenetic trees and evolutionary rates.

**Figure 1.**
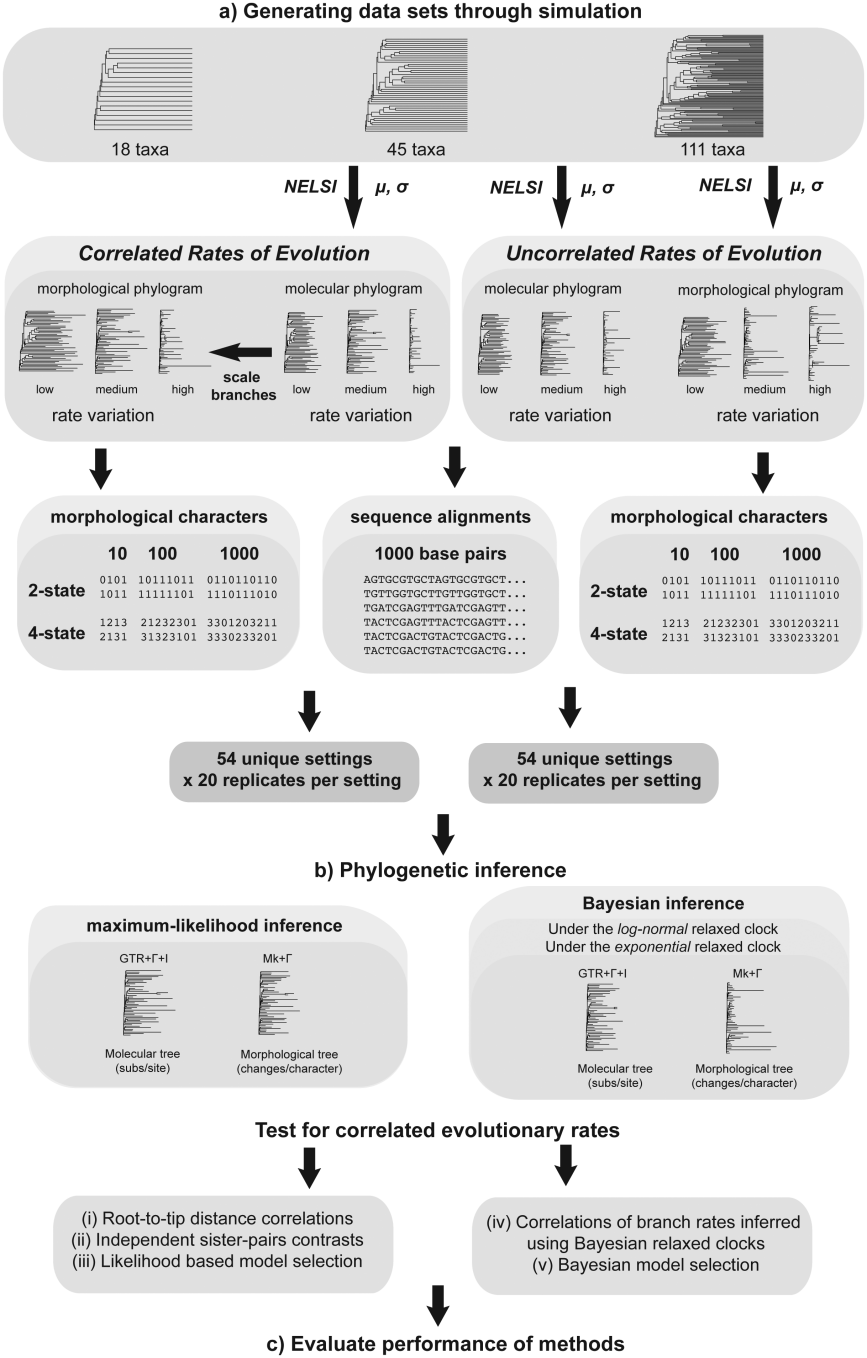
Flowchart of simulation study. a) Generating data sets through simulation. Molecular phylograms are generated using chronograms with 3 tree sizes (18, 45, or 111 taxa), with a mean rate of evolution (*μ*) that has been inferred from an empirical data set, and standard deviation (*σ*) representing 3 levels of among-lineage rate variation (0.25, 0.75, or 1.25). The branch lengths of the molecular phylogram are then multiplied by an empirical scaling factor to obtain a morphological phylogram with correlated rates of evolution along each branch. To obtain trees without correlated rates of evolution, morphological phylograms are simply generated using an independent mean rate of evolution (*μ*), but on the same chronogram and with the same standard deviation (*σ*) as the molecular phylogram. Simulations were performed under a total of 54 distinct settings each for the scenarios with correlated and uncorrelated rates of evolution, with 20 replicates per setting. b) Phylogenetic inference. Using a fixed tree topology, molecular and morphological branch lengths were inferred using maximum-likelihood and Bayesian analyses. When inferring trees using Bayesian analyses, the trees were inferred under the lognormal relaxed clock as well as the exponential relaxed clock, to test the impact of model mismatch. Marginal likelihoods were estimated for the purpose of Bayesian model selection. Five methods were used to test for correlated rates of evolutionary change, (*i*) root-to-tip distance correlations, (*ii*) independent sister-pairs contrasts, (*iii*) likelihood-based model selection, (*iv*) correlations of Bayesian branch rates, and (*v*) Bayesian model selection. c) Evaluate performance of methods. We compared the accuracy and power of the 5 methods of testing for correlated rates of evolutionary change.

We performed simulations using chronograms (branch lengths measured in millions of years, Myr) of 3 different sizes, based on a 792-species angiosperm tree from Magallón et al. (2015) used by Sauquet et al. (2017). From this tree, we sampled 18, 45, or 111 species to represent diverse angiosperm lineages (Fig. 1a). These 3 trees had root ages of 139.40 Ma (not including the outgroup *Welwitschia mirabilis)*.

We rescaled the branch lengths of the chronograms to produce phylograms (branch lengths measured in substitutions/site). To do this, we used the R package *NELSI* (Ho et al. 2015) to generate branch lengths according to an uncorrelated lognormal clock (Drummond et al. 2006). The branch rates had a mean of 9.65 × 10^−4^ subs/site/Myr, inferred in a previous analysis of 2 nuclear markers (18S and 26S ribosomal DNA) and 3 plastid protein-coding genes (*atpB*, *rbcL*, and *matK*) from 792 angiosperm species (Magallón et al. 2015). We generated branch rates with low, moderate, and high levels of variation, with respective standard deviations of 0.25, 0.75, and 1.25.

We then performed simulations under 2 scenarios, in which molecular and morphological evolutionary rates were either correlated or uncorrelated. To generate a morphological phylogram with branch rates correlated with those of the molecular phylogram, we scaled the branch lengths of the latter by a factor of 1.90 (Fig. 1a). This scaling factor was based on a mean rate of morphological evolution of 1.83 × 10^−3^ changes/character/Myr, inferred from 27 floral characters for 792 angiosperm species (Sauquet et al. 2017).

For our simulations with branch rates being uncoupled between molecular and morphological data sets, we simply used *NELSI* to generate independent sets of branch rates and used these to rescale the branch lengths of the chronograms (Fig. 1a). We used the same mean evolutionary rates and standard deviations for the simulations with uncorrelated rates as we did for the synthetic data sets with correlated rates, which are described above.

#### Generating molecular sequence alignments and morphological character matrices.

We used Seq-Gen version 1.3.4 (Rambaut and Grassly 1997) to simulate the evolution of nucleotide sequences on the phylograms produced by the previous step (Fig. 1a). These simulations produced sequence alignments with lengths of 1000 nucleotides, reflecting typical sizes of nuclear and plastid protein-coding genes. The generating model was the general time-reversible (GTR) model, with gamma-distributed rate heterogeneity (+Γ) and a proportion of invariant sites (+I). The nucleotide transition rates, nucleotide frequencies, and gamma shape parameters were based on estimates from Magallón et al. (2015) and are listed in the Supplementary Material.

We generated morphological data matrices consisting of 10, 100, and 1000 characters (Fig. 1a). Matrices with either 2-state (binary) or 4-state characters were simulated. Character evolution was simulated using the R package *geiger* (Pennell et al. 2014) under the Mk model (Lewis 2001), a generalization of the Jukes-Cantor (1969) model of molecular evolution. The relative rate for each character was drawn randomly from a gamma distribution with a shape parameter of 1.39 inferred via maximum-likelihood analysis of the floral character data set from Schönenberger et al. (2020), comprising 30 binary and multi-state morphological characters scored for 792 extant angiosperm species (see Supplementary Material). Approximately 33.0% of the data set comprises missing data or characters that were polymorphic within species. This morphological data set was also used in the empirical analyses in this study.

#### Summary of simulation scenarios.

The settings described above yielded a total of 54 distinct simulation scenarios. Our simulations included trees of 3 sizes (18, 45, and 111 taxa). Nucleotide sequences consistently had a length of 1000 nucleotides, but we varied the number of morphological characters (10, 100, and 1000 characters) and the number of possible morphological character states (either 2- or 4-state characters). We simulated 3 levels of among-lineage rate heterogeneity with standard deviations of 0.25, 0.75, and 1.25 for the molecular and morphological data sets (Fig. 1a). For each of the 54 simulation settings, we generated 20 replicate data sets. Thus, our simulations produced a total of 1080 pairs of molecular and morphological data sets for each of the “correlated” and “uncorrelated” settings.

### Phylogenetic Inference

#### Maximum-likelihood inference.

We inferred phylograms from the simulated molecular and morphological data sets using maximum likelihood in IQ-TREE2 (version 2.0.6; Bui et al. 2020) (Fig. 1b). In each analysis, the tree topology was constrained to match that used for simulation, with the addition of 1 outgroup taxon, the gymnosperm *Welwitschia mirabilis*. Analyses of the molecular data used the general-time-reversible model, with gamma-distributed rates (4 discrete categories) and invariant sites (GTR+Γ+I). Analyses of the morphological data used the time-homogeneous Mk model and gamma rate heterogeneity with 4 discrete categories (Mk+Γ) (Supplementary Material). Following maximum-likelihood inference, the gymnosperm outgroup was removed from each tree. The branch lengths from these phylograms were used for root-to-tip distance correlations, independent sister-pairs contrasts, and likelihood-based model selection, which are described in the next section.

#### Bayesian inference.

We analyzed the molecular and morphological data using Bayesian inference in BEAST2 version 2.6.6 (Bouckaert et al. 2019) (Fig. 1b). In each analysis, the tree topology was constrained to match that used for simulation. Molecular and morphological data were partitioned so that they were assigned distinct substitution models, with the GTR+Γ+I model for the molecular data set and the Mk+ Γ model for the morphological data set. The molecular and morphological data sets were assigned separate lognormal relaxed clocks, with the rate of morphological evolution fixed to 1 and the relative rate of molecular evolution being estimated. The molecular and morphological data subsets shared the same fixed tree topology, with a birth-death tree prior for the relative node times. We also performed an analysis in which molecular and morphological data shared the same lognormal relaxed clock, to allow comparison between linked and unlinked clock models using Bayesian model selection.

For each Bayesian analysis, the posterior distribution was estimated using Markov chain Monte Carlo (MCMC) sampling. We ran 2 independent chains, each of 10,000,000 steps, with samples drawn every 1000 steps. The first 10% of samples were discarded as burn-in. After checking for convergence and sufficient sampling (effective sample sizes of parameters greater than 200) using the LogAnalyzer function, we combined the samples from the 2 chains. The tree samples were summarized using TreeAnnotator, part of the BEAST2 package. We obtained the estimates of branch rates from the summarized trees and used them in our tests of rate correlations, described below.

### Testing for Correlated Rates of Evolution

We evaluated the performance of 5 methods for testing for correlated evolutionary rates: (*i*) root-to-tip distance correlations, (*ii*) independent sister-pairs contrasts, (*iii*) likelihood-based model selection, (*iv*) correlations of Bayesian branch rates, and (*v*) Bayesian model selection (Fig. 1b). The first 3 methods were performed on maximum-likelihood trees that were inferred from the synthetic data. The last 2 methods were performed using the results of our Bayesian phylogenetic analyses.

#### (i) Root-to-tip distance correlations.

The first method of testing for correlated rates was based on examination of the root-to-tip distances in the maximum-likelihood phylograms. For each matched pair of molecular and morphological phylograms, we calculated patristic root-to-tip distances using the distRoot function in the R package *adephylo* (Jombart et al. 2010) and tested for a correlation between the molecular and morphological root-to-tip distances. To address nonindependence among the root-to-tip distances, we calculated the *P*-value using a permutation test in the R package *jmuOutlier* (Garren 2017; Higgins 2004), with 20,000 replicates.

#### (ii) Independent sister-pairs contrasts.

The second method of testing for correlated rates involved taking phylogenetically independent pairs of taxa (sister pairs) and comparing their relative branch lengths between the molecular and morphological phylograms. We selected sister species that shared a most recent common ancestor to the exclusion of other sister pairs, which avoids the problem of phylogenetic nonindependence but reduces the amount of data (Felsenstein 1985; Bromham et al. 2002). We used the R package *diverge* to extract sister pairs for each tree (Anderson and Weir 2020). Lists of sister pairs for each tree size are provided in the Supplementary Material. By definition, the 2 branches in each sister pair have been evolving for the same amount of time, such that their phylogram length reflects their relative evolutionary rate. We computed the difference between the branch lengths of sister species in the molecular and morphological phylograms inferred using maximum likelihood. We tested for correlations between the molecular and morphological contrasts using the nonparametric Spearman’s rank correlation test, to allow for violations of bivariate normality and homoscedasticity. The contrasts were log-transformed and standardized following standard guidelines by dividing by the square root of the time since divergence between sister species (see Supplementary Materials for details; Garland et al. 1992; Freckleton 2000; Welch and Waxman 2008).

#### (iii) Likelihood-based model selection.

The third method of testing for correlated rates involved likelihood-based model selection using information criteria. We analyzed the molecular and morphological data using maximum likelihood in IQ-TREE2 and compared models in which the branch lengths were either linked (“proportionate”) or unlinked between the molecular and morphological trees. These 2 models reflect correlated and uncorrelated branch rates, respectively, between molecular and morphological data (Duchêne et al. 2020). We compared the models using the corrected Akaike information criterion (AICc) and the Bayesian information criterion (BIC), following the guidelines outlined by Lotterhos et al. (2022).

#### (iv) Correlations of Bayesian branch rates.

The fourth method of testing for correlated rates was based on the inferred branch rates from Bayesian phylogenetic analyses. We tested for correlations between the branch rates inferred using the lognormal relaxed clock for the molecular and morphological data sets. To compute the significance of the correlation, we used Spearman’s rank-order correlation test to avoid violations of bivariate normality and homoscedasticity. For comparison, tests were performed using the mean and median posterior branch rates. The mean posterior rate is commonly reported in Bayesian phylogenetic analyses, but the median rate might be more appropriate because the marginal posterior distributions are skewed. Furthermore, to evaluate the impact of removing poorly estimated branch rates, which might be due to the limited number of morphological characters under some of the simulation settings, we also tested correlations using mean and median posterior branches after removing the chronologically shortest 50% of branches.

#### (v) Bayesian model selection.

The fifth method of testing for correlated rates involved the use of Bayesian model selection to compare the support for linked or unlinked branch rates between molecular and morphological data. Model selection was performed using Bayes factors (BFs), which compare the marginal likelihoods of the 2 models. The marginal likelihoods of the linked and unlinked relaxed-clock models were estimated using the “MS” Model Selection package in BEAST2. We used generalized-stepping-stone sampling (Baele et al. 2016), with 25 steps and chain lengths of 400,000. The ratio of the marginal likelihoods was used to compute the BF, which was then interpreted using the guidelines of Kass and Raftery (1995).

#### Evaluation of performance.

We used 2 approaches to evaluate the performance of the 5 methods of testing for rate correlations. First, we evaluated the methods by their power, which is the ability to detect positive correlations under the largest number of scenarios. Second, we examined the accuracy of the methods, regarded here as the ability to detect positive correlations without false positives. We do not attempt to evaluate the ability of the methods to infer the correct *degree* of rate correlation, that is, the correlation coefficient.

#### Impacts of model mismatch.

Evolutionary models are rarely able to capture the complexity of real evolutionary processes, so there is almost invariably some degree of model mismatch in practice. To evaluate the impact of model mismatch, we performed additional Bayesian analyses in which we inferred branch rates using exponential relaxed clocks (Drummond et al. 2006), which depart from the lognormal relaxed clocks that were used as the generating model. These analyses were used to test the accuracy and power of correlations of Bayesian branch rates and Bayesian model selection. All other settings matched those in our analyses described above.

### Case Study: Flowering Plants

To test for correlated rates of molecular and morphological evolution in empirical data, we applied the above 5 methods to large angiosperm data sets. Molecular data were obtained from the One Thousand Plant Transcriptomes Initiative (2019). This data set includes nucleotide sequences from 410 protein-coding nuclear genes from 1124 green plants, glaucophytes, and red algae. For computational tractability, we analyzed a subset of 111 angiosperm species and 1 gymnosperm outgroup (*Welwitschia mirabilis*), matching the angiosperm species in the morphological data set. We applied the data-partitioning scheme as outlined by the One Thousand Plant Transcriptomes Initiative (2019), comprising 8 data subsets, and estimated branch lengths on a fixed tree topology using maximum-likelihood analysis and Bayesian inference. The substitution model for each data subset was selected using ModelFinder in IQTREE2 (Kalyaanamoorthy et al. 2017).

The morphological data set was sourced from the eFLOWER project (Sauquet et al. 2017). Here we used the latest version from the Schönenberger et al. (2020) data set, exported from the PROTEUS database on 17 May 2021. This continually curated data set represents an expanded set of 30 floral characters for 792 angiosperm species, initially published by Sauquet et al. (2017). These are discrete binary and multi-state morphological characters, including features such as the structural sex of flowers, ovary position, phyllotaxy, number of reproductive parts, and fusion of ovaries. The number of characters scored is smaller than those of morphological matrices available for animals; for example, the data set analyzed by O’Leary et al. (2013) comprises 2954 traits recorded for mammals. However, the eFLOWER data set features the greatest taxonomic sampling for angiosperms. We used a subset of 111 angiosperm species for our analyses and partitioned the data according to the number of character states (i.e., 2-, 3-, 4-, and 5-state data were treated as separate subsets). Branch lengths were estimated on a fixed tree topology using maximum-likelihood analysis and Bayesian inference, using the Mk+Γ model of evolution with correction for ascertainment bias.

We performed Bayesian phylogenetic analyses of the genomic data and floral characters with and without calibrations. In the former case, we implemented secondary calibrations on the ages of key angiosperm groups. The secondary calibrations were sourced from Magallón et al. (2015) and applied as normal priors on node ages (Ho and Phillips 2009). To estimate the posterior distribution, we sampled from 5 independent MCMC runs each with a chain length of 50 million steps. Samples were drawn every 5000 steps. After checking for convergence and sufficient sampling in Tracer, we removed a burn-in fraction of 10%, leaving a total of 45,000 sampled trees. Further details of the angiosperm case study, including settings and secondary calibrations, are available in the Supplementary Material.

## Results

### Performance in Detecting Correlated Evolutionary Rates

**Figure 2.**
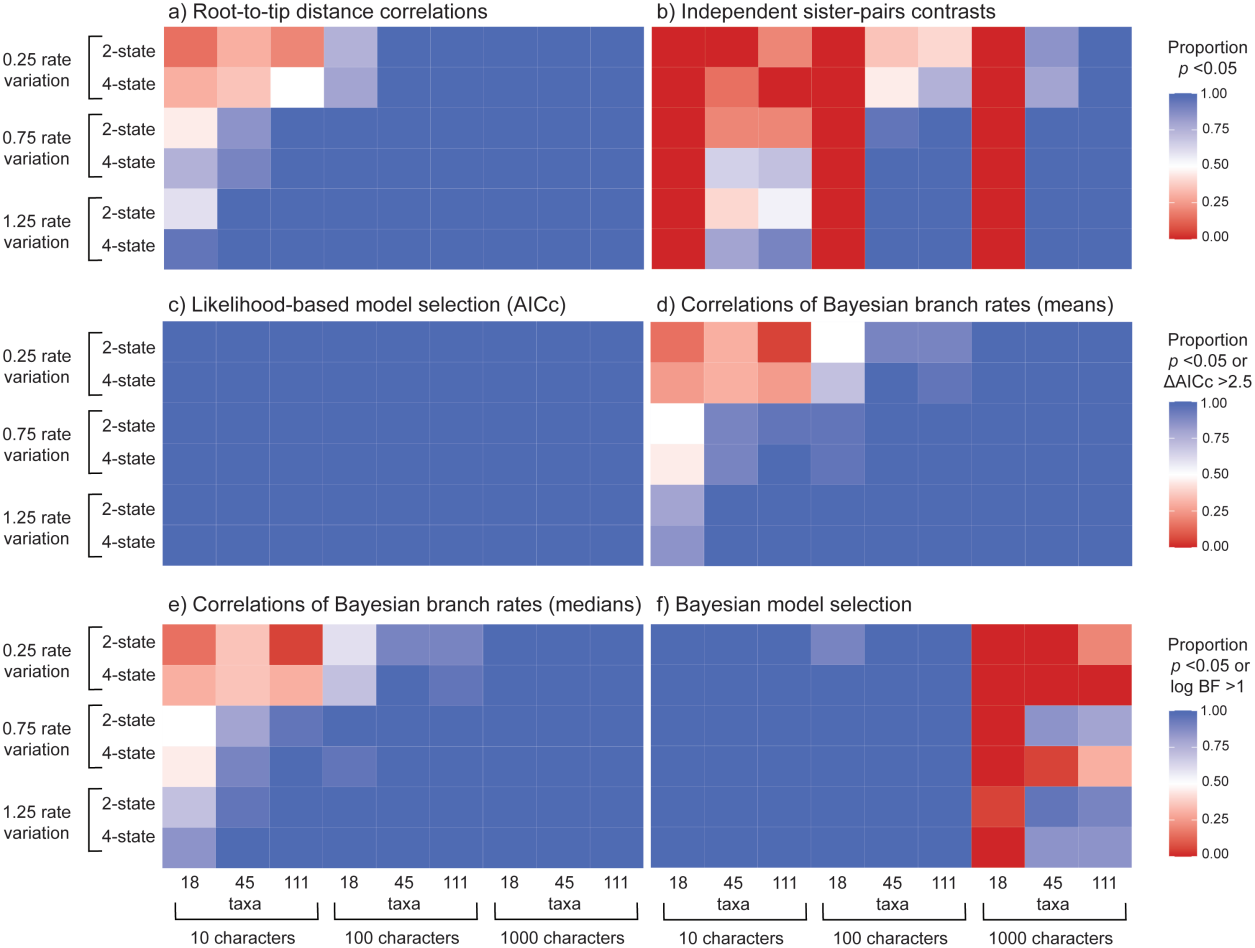
Heatmaps showing the performance of 5 approaches for testing for correlations between molecular and morphological evolutionary rates, for data produced by simulation with correlated evolutionary rates: a) root-to-tip distance correlations; b) independent sister-pairs contrasts; c) likelihood-based model selection using the corrected Akaike information criterion (AICc); correlations of Bayesian branch rates using d) mean posterior branch rates of branches or e) median posterior branch rates; and f) Bayesian model selection. Cooler colors denote (correct) detection of rate correlations. In each panel, rows give results under 6 scenarios, representing combinations of 3 levels of among-lineage rate variation [0.25, 0.75, 1.25], and either 2- or 4-state morphological characters. In each panel, columns give results for data sets of various sizes, representing combinations of 3 numbers of morphological characters [10, 100, 1000] and 3 numbers of taxa [18, 45, 111]. For methods (a)–(b) and (d)–(e), colors indicate the proportion of 20 replicates for each setting that yielded a significant rate correlation (i.e., *P* < 0.05). For method (c), colors indicate the proportion of 20 replicates for each setting that yielded ΔAICc > 2.5, supporting a model of linked rates over a model of unlinked rates. For method (f), colors indicate the proportion of 20 replicates for each setting that yielded a log BF > 1.0 for a model of linked rates over a model of unlinked rates. For heatmaps of likelihood-based model selection using the BIC, and correlations of Bayesian branch rates with the 50% chronologically shortest branches removed, see Supplementary Material.

**Figure 3.**
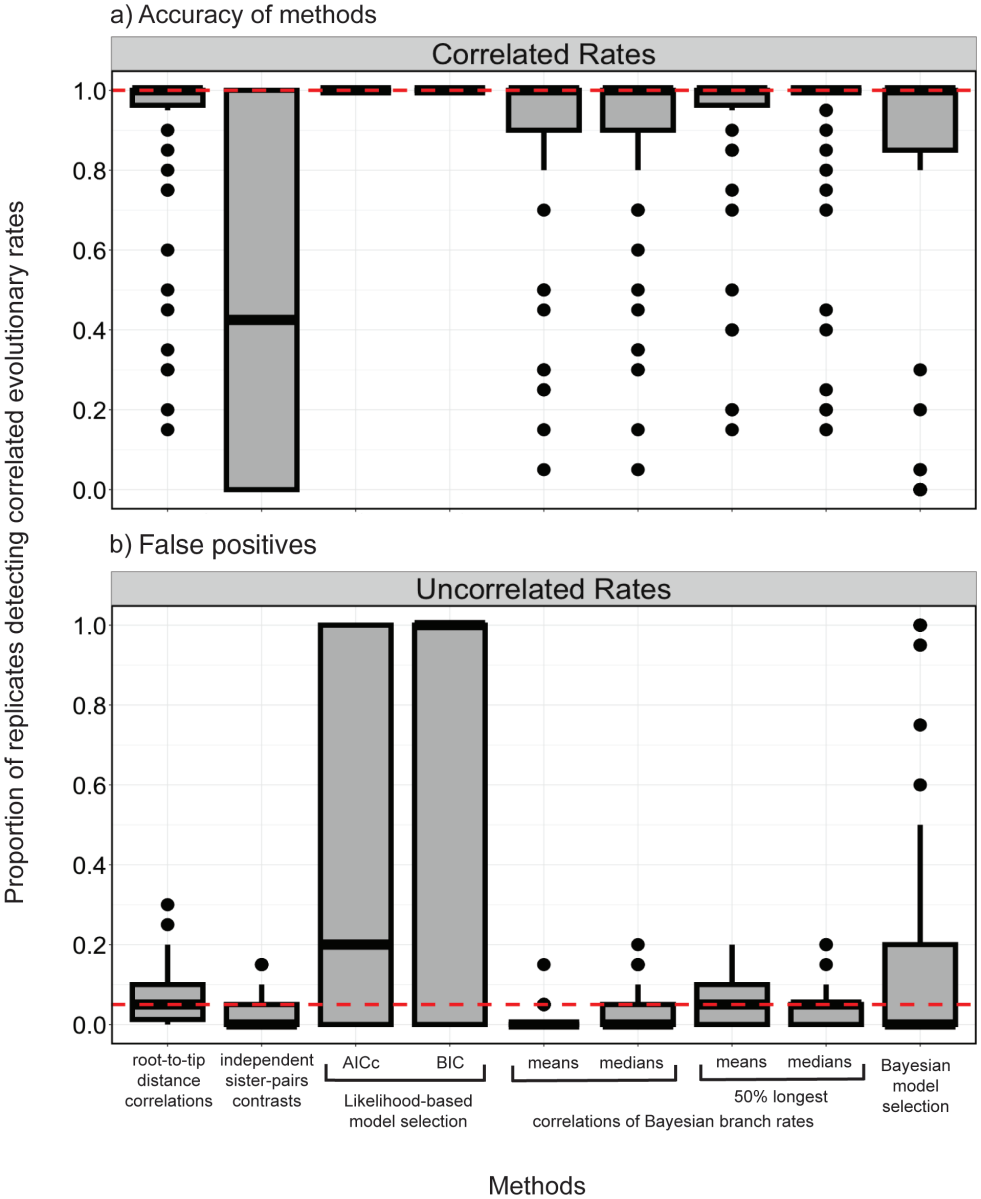
Boxplots showing the frequency of detecting correlated rates of evolution between simulated molecular and morphological data using different methods. a) Accuracy of the methods when analyzing data simulated with correlated rates. The dashed horizontal line represents the ideal detection of correlated rates of evolution (100% of scenarios). b) Propensity of methods to detect correlations when analyzing data simulated with uncorrelated rates (false positives). The dashed horizontal line represents the detection of correlated rates of evolution expected under frequentist statistics with a critical value of 0.05 (false-positive rate of 5%).

Using data generated by simulation, we compared 5 methods for testing for correlations in evolutionary rates between molecular sequences and morphological characters. Likelihood-based model selection invariably detected correlated evolutionary rates across scenarios (100% for both AICc and BIC) but had an unacceptably high frequency of false positives (Figs. 2 and 3). Analyses of branch rates from Bayesian relaxed-clock inference were able to detect correlated rates between molecular and morphological data under a wide range of simulation settings (Figs. 2 and 3). Under this method, the mean and median posterior branch rates were equally effective, with an average detection of correlated evolutionary rates of 86.1% and 86.3%, respectively. After removing the chronologically shortest 50% of branches, both methods performed slightly better, with the average detection of correlated rates increasing to 90.3% and 89.9% (Supplementary Material). The performance of this method was closely followed by root-to-tip distance correlations (88.6%), Bayesian model selection (77.2%), and independent sister-pairs contrasts (48.7%).

**Figure 4.**
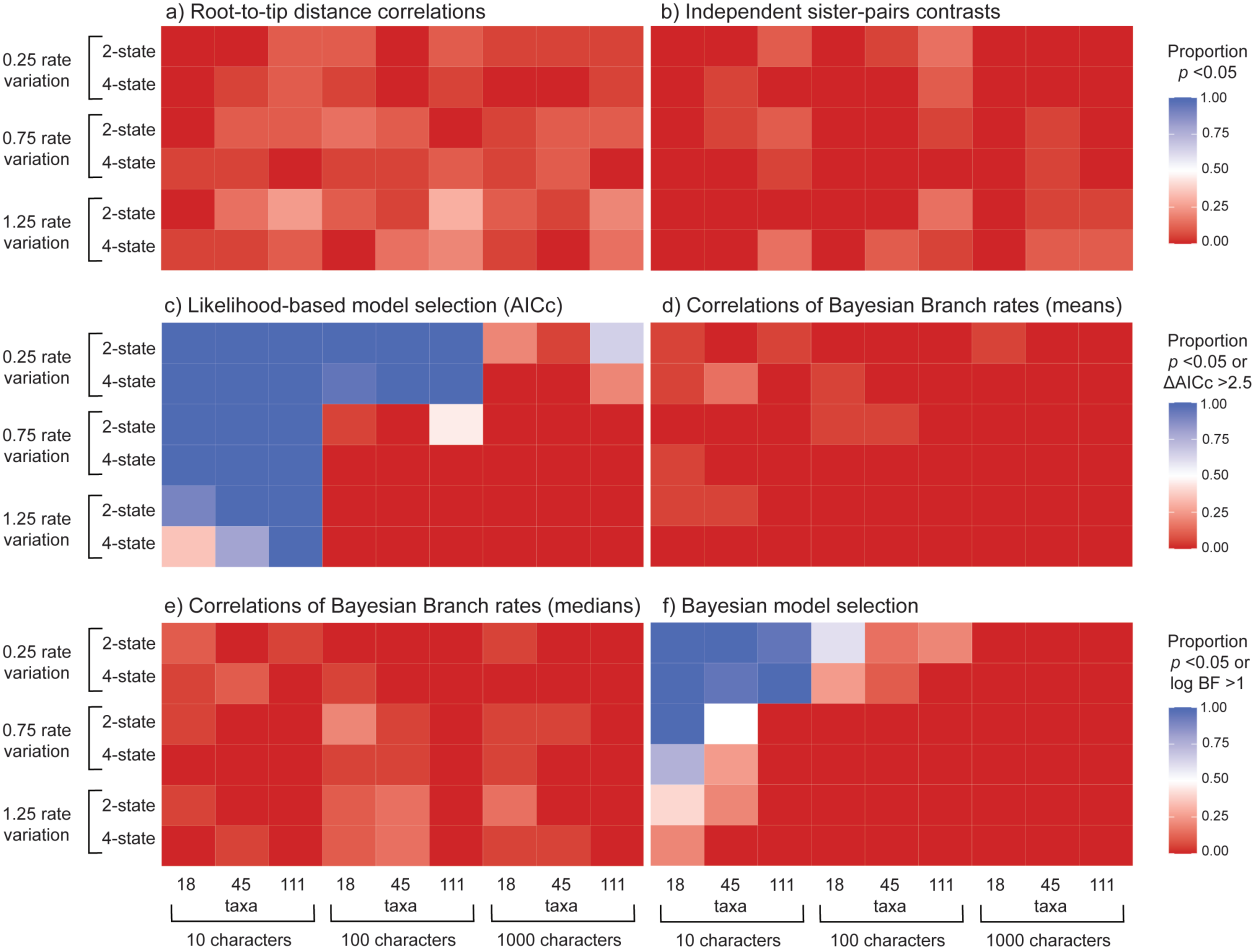
Heatmaps showing the performance of 5 approaches for testing for correlations between molecular and morphological evolutionary rates, for data produced by simulation without correlated evolutionary rates: a) root-to-tip distance correlations; b) independent sister-pairs contrasts; c) likelihood-based model selection using the corrected Akaike information criterion; correlations of Bayesian branch rates using d) mean posterior branch rates or e) median posterior branch rates; and f) Bayesian model selection. Cooler colors denote (incorrect) detection of rate correlations. In each panel, rows give results under 6 scenarios, representing combinations of 3 levels of among-lineage rate variation [0.25, 0.75, 1.25], and either 2- or 4-state morphological characters. In each panel, columns give results for data sets of various sizes, representing combinations of 3 numbers of morphological characters [10, 100, 1000] and 3 numbers of taxa [18, 45, 111]. For methods (a)–(b) and (d)–(e), colors indicate the proportion of 20 replicates for each setting that yielded a significant rate correlation (i.e., *P* < 0.05). For method (c), colors indicate the proportion of 20 replicates for each setting that yielded ΔAICc > 2.5, supporting a model of linked rates over a model of unlinked rates. For method (f), colors indicate the proportion of 20 replicates for each setting that yielded a log BF > 1.0 for a model of linked rates over a model of unlinked rates. For heatmaps of likelihood-based model selection using the BIC, and correlations of Bayesian branch rates with the 50% chronologically shortest branches removed, see Supplementary Material.

When we analyzed molecular and morphological data sets that had been generated without correlated rates, we found low false-positive rates when using correlations of Bayesian branch rates (1.20% and 3.33% for mean and median posterior branch rates, respectively; 5% and 4.62% after removing the shortest 50% of branches), root-to-tip distance correlations (7.13%), and independent sister-pairs contrasts (2.87%) (Figs. 3 and 4). However, both likelihood-based and Bayesian model selection yielded a high frequency of false positives when using either the AICc (45.6%) or BIC (65.4%), and 19.4% using BFs.

### Impacts of Varying Simulation Conditions

**Figure 5.**
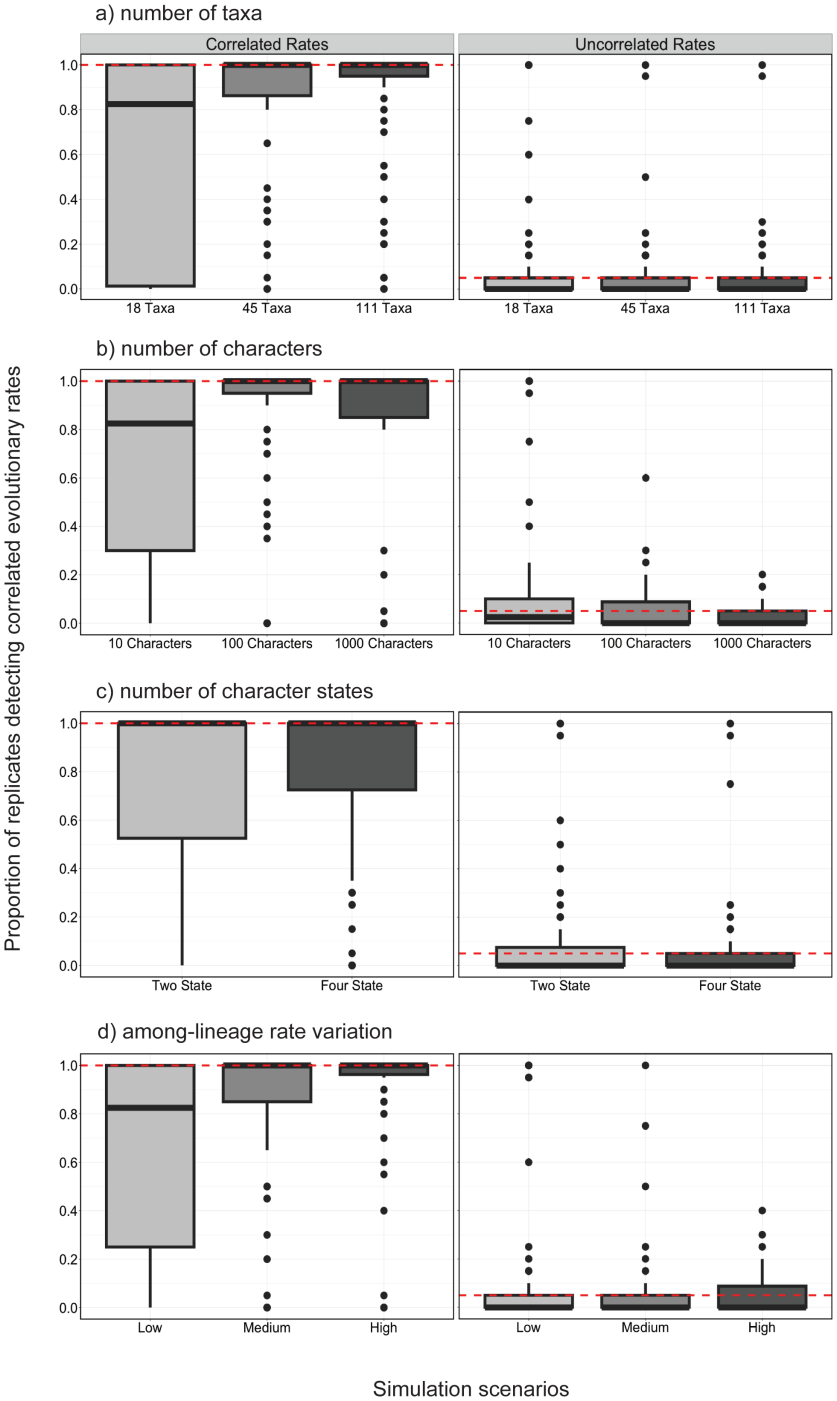
Boxplots showing the frequency of detecting correlated rates of evolution between simulated molecular and morphological data using different settings. The left panels show the performance of methods when data were simulated with correlated rates, with dashed horizontal lines representing the ideal detection of correlated rates of evolution (100% of scenarios). The right panels show results when simulated with uncorrelated rates, with dashed horizontal lines representing the detection of correlated rates of evolution expected under frequentist statistics with a critical value of 0.05 (false-positive rate of 5%). The different settings used were: a) 3 tree sizes, b) 3 sizes of morphological character matrices, c) 2 numbers of possible morphological character states, and d) 3 levels of among-lineage rate variation. The results were pooled across all methods except for likelihood-based model selection, since this method had such a high rate of false positives and would unreasonably skew the detection of correlations. Correlations of Bayesian branch rates excluding the 50% shortest branches are not shown. Boxplots calculated with the results for each of the 5 methods separately are included in the Supplementary Material.

We tested the effect of tree size, including 18, 45, and 111 taxa in the simulations, to evaluate its impact on the ability to detect correlated evolutionary rates. Given the propensity of likelihood-based model selection to consistently detect both genuine correlations and false positives, the statistics that we report have excluded this method. The number of taxa influenced the detection of correlations predictably (Fig. 5a), with performance increasing with tree size (61.1%, 84.7%, and 86.3% for tree sizes of 18, 45, and 111 taxa, respectively). Generally, analyses of the 18 taxon-set performed poorly when there were only 10 morphological characters (Fig. 2). This was especially apparent for independent sister-pairs contrasts, where analyses of 18-taxon data sets failed to detect rate correlations regardless of the degree of among-lineage rate variation, number of character states, and numbers of morphological characters.

We varied the number of morphological characters [10, 100, 1000] to evaluate their impact on the ability of the 5 methods to detect correlated evolutionary rates. We found that the average detection of positive correlations increased from 64.8%, 88%, to 79.3% for data sets with 10, 100, and 1000 morphological characters, respectively (Figs. 2 and 5b). The effect depended on the amount of among-lineage rate variation; where branch rates had a standard deviation of at least 0.75, the 4 best approaches were generally able to detect correlations with any number of morphological characters (Fig. 2). However, when there was a low degree of among-lineage rate variation, rate correlations could not be detected when there were only 10 morphological characters. Furthermore, likelihood-based model selection produced a high rate of false positives, but this was mitigated when there were either 100 or 1000 morphological characters and moderate or high among-lineage rate variation (Fig. 4c; Supplementary Material). In contrast, when there were 1000 morphological characters, Bayesian model selection frequently failed to detect correlated rates of evolution (Fig. 2f).

The number of character states for the morphological data had a minor impact on detection of correlations. Generally, correlations were more frequently detected when the morphological data comprised 4-state characters, with a positive detection of 76.1% compared with 78.6% when the data comprised 2-state characters (Figs. 2 and 5c). However, this effect was negligible when there were greater than 10 morphological characters and 18 taxa in the data set (Fig. 2).

Across the 4 most powerful and accurate methods, the most important factor for detection of correlated evolutionary rates was among-lineage rate variation (Figs. 2 and 5d). When we simulated low among-lineage rate variation in the data sets, correlated rates could only be detected across 62.4% of replicates, whereas medium and high levels of rate variation increased detection to 82% and 87.7%, respectively. Where there was low among-lineage rate variation, with branch rates having a standard deviation of 0.25, the 4 best approaches were generally unable to detect correlations without sampling at least 100 morphological characters (Fig. 2; Supplementary Material). This was especially true for Bayesian model selection, which could not detect correlated rates of evolution at the lowest level of among-lineage rate variation.

### Impacts of Model Mismatch

**Figure 6.**
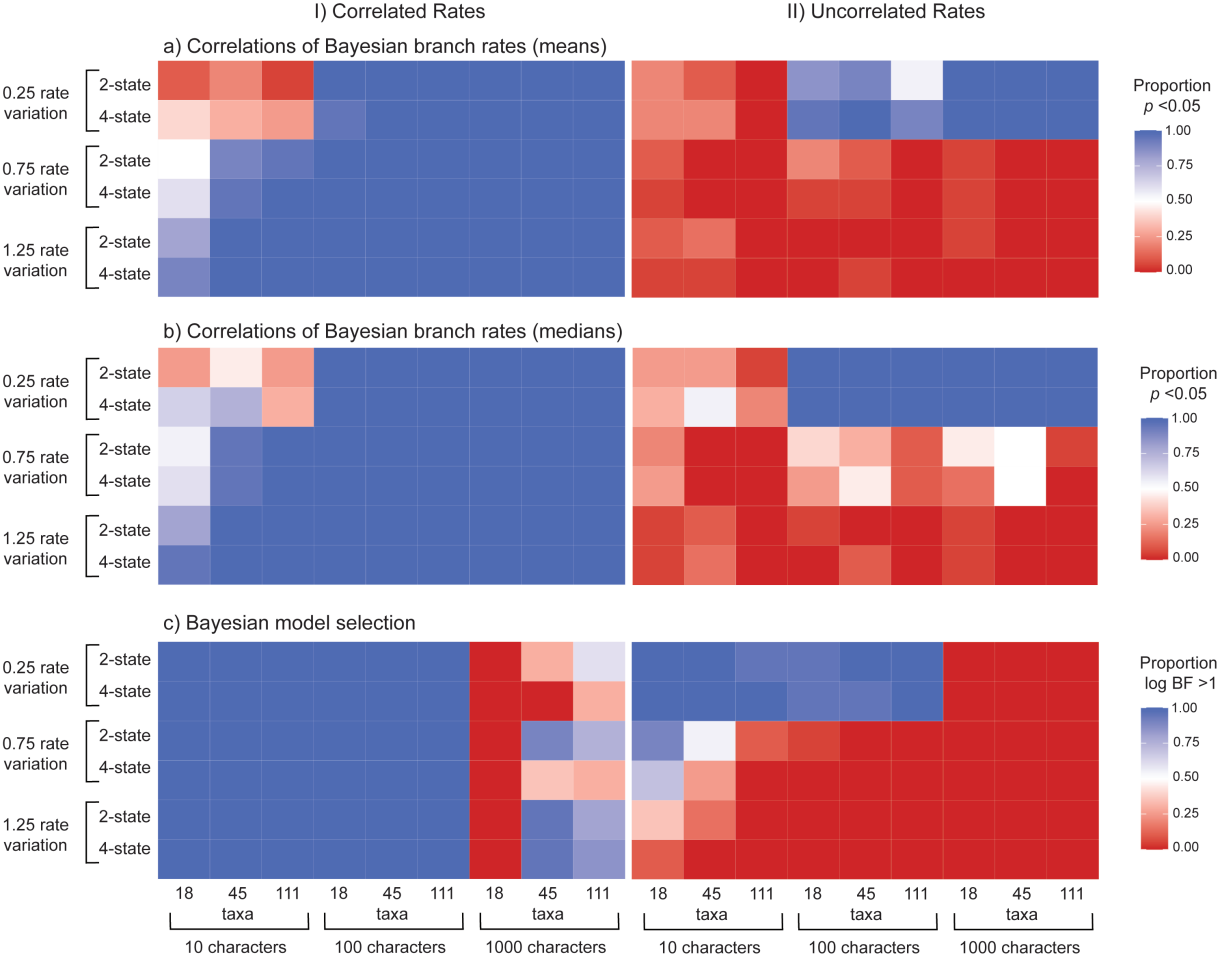
Heatmaps showing the performance of 3 approaches for testing for correlations between molecular and morphological evolutionary rates, for data produced by simulation with I) correlated and II) uncorrelated morphological–molecular evolutionary rates using an exponential relaxed clock for analyses. a) correlations of Bayesian branch rates using mean posterior branch rates or b) median posterior branch rates, and c) Bayesian model selection. Cooler colors denote detection of rate correlations. In each panel, rows give results under 6 scenarios, representing combinations of 3 levels of among-lineage rate variation [0.25, 0.75, 1.25], and either 2- or 4-state morphological characters. In each panel, columns give results for data sets of various sizes, representing combinations of 3 numbers of morphological characters [10, 100, 1000] and 3 numbers of taxa [18, 45, 111]. For methods (a)–(b) colors indicate the proportion of 20 replicates for each setting that yielded a significant rate correlation (i.e., *P* < 0.05). For method (c), colors indicate the proportion of 20 replicates for each setting that yielded a log BF > 1.0 for a model of linked rates over a model of unlinked rates. For heatmaps of correlations of Bayesian branch rates with the 50% chronologically shortest branches removed, see Supplementary Material.

**Figure 7.**
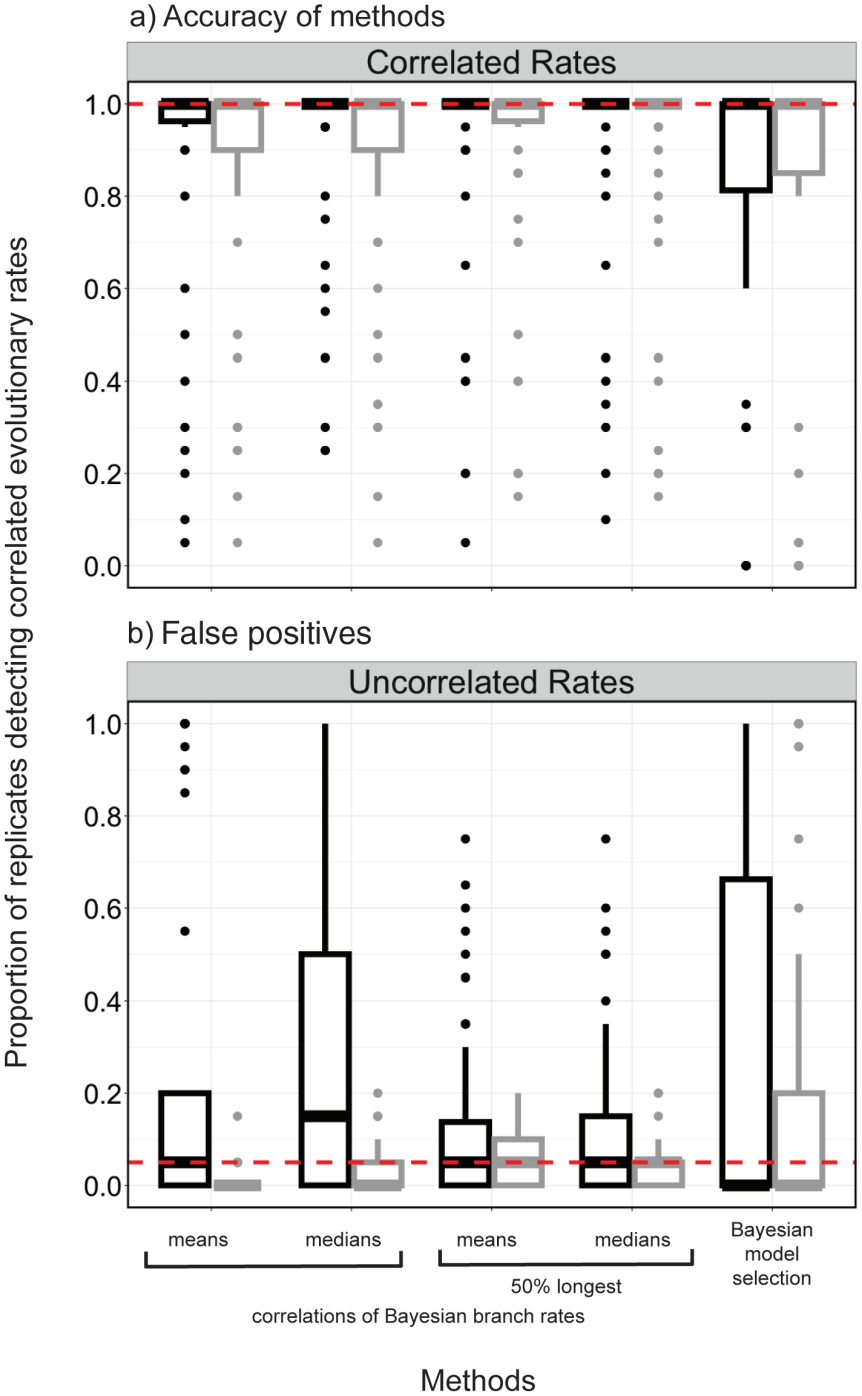
Boxplots showing the frequency of detecting correlated rates of evolution between simulated molecular and morphological data using different methods. a) Accuracy of the methods when analyzing data simulated with correlated rates. The dashed horizontal line represents the ideal detection of correlated rates of evolution (100% of scenarios). b) Propensity of methods to detect correlations when analyzing data simulated with uncorrelated rates (false positives). The dashed horizontal line represents the detection of correlated rates of evolution expected under frequentist statistics with a critical value of 0.05 (false-positive rate of 5%). For each method, results are shown for analyses of 2 different scenarios: where model mismatch was implemented, by analyzing synthetic data using the exponential relaxed clock (shown in black) and where the analyzing model matched the generating model, the lognormal relaxed clock (shown in gray).

We explored the impacts of model mismatch by performing Bayesian inference with an exponential relaxed clock rather than a lognormal relaxed clock. Bayesian inference of evolutionary rates was generally robust to the choice of clock model; analyses of the branch rates were able to detect correlated rates across many settings (Figs. 6 and 7; Supplementary Material). The mean and median posterior branch rates were similarly effective at detecting correlations; with an average detection of 88.6% and 91.6%, respectively. This method failed to detect correlated rates when there were 10 morphological characters in the synthetic data sets and a low amount of rate variation among lineages (Fig 6; Supplementary Material). Bayesian model selection was less powerful, with an average detection of 79.7%. These rates of correct detection were comparable to the analyses with the relaxed lognormal clock, where the models were matched. As seen in the model-matched analyses, correlations were easier to detect when tree sizes and morphological character data sets were larger (Fig. 6; Supplementary Material).

The rate of false positives for the analyses with model mismatch was much higher (Figs. 6 and 7; Supplementary Material); correlations of Bayesian branch rates had a false-positive detection rate of 24.0% for mean posterior branch rates and 33.1% for median posterior branch rates, and Bayesian model selection had a false-positive rate of 27.7%. However, after removing the chronologically shortest 50% of branch rates, the false-positive rate dropped to 14.0% when using both mean and median posterior branch rates. The high false-positive rate when analyzing the data using a mismatched model was particularly pronounced when there was a low degree of rate variation among lineages (Fig. 6d–f; Supplementary Material).

### Case Study: Flowering Plants

**Figure 8.**
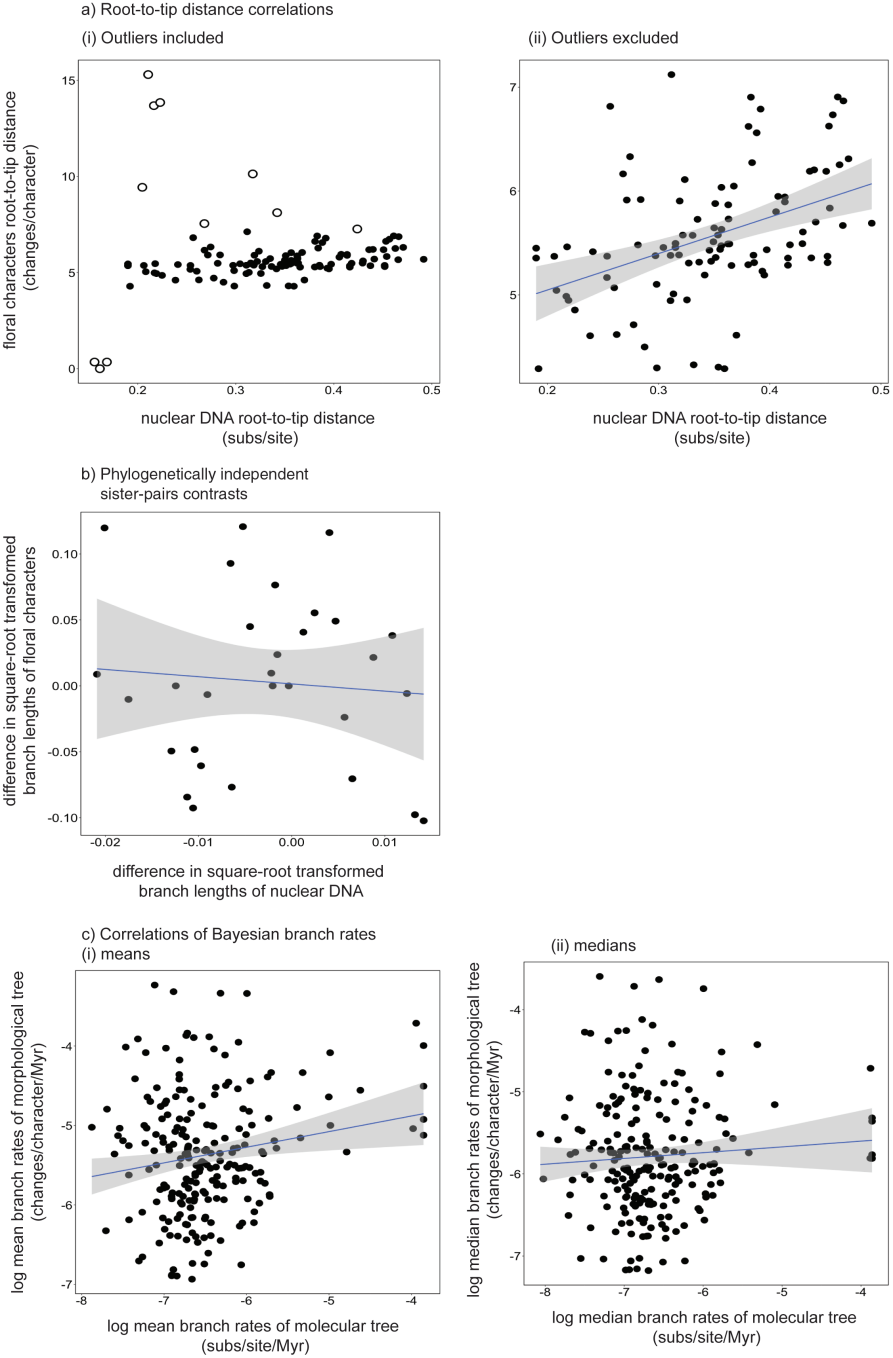
Comparisons between evolutionary rates of nuclear genomic DNA and floral characters inferred from angiosperms. The methods used to test for correlated rates of evolution are a) root-to-tip distance correlation with (i) outliers shown in white circles and (ii) the outliers excluded; b) phylogenetically independent sister-pairs contrasts; c) correlations of Bayesian posterior branch rates using (i) mean and (ii) median posterior branch rates. The plot for each comparison has been fit with a linear model, which is displayed along with the 95% confidence interval.

In our analyses of genomic DNA and floral characters in angiosperms, we found that 2 of the 5 methods, root-to-tip distance correlations and likelihood-based model selection, detected a correlation in evolutionary rates. We found evidence of a correlation in our analysis of root-to-tip distances (*P* = 5 × 10^−6^; Fig. 8a). Outliers (data points outside 1.5 times the interquartile range) were excluded from the permutation test, but root-to-tip distances were significantly correlated both before and after removal of outliers (see Supplementary Material; Fig 8b). These outliers included the branch leading to the sister taxon to all remaining angiosperms, *Amborella trichopoda*, which had a low morphological evolutionary rate of 2.38 × 10^−6^ changes/character. Other ANA-grade angiosperms, such as *Austrobaileya scandens* and *Illicium floridanum*, similarly had low rates of morphological change and were removed from the test (Supplementary Material). Likelihood-based model selection also yielded very strong support for linking branch lengths between nuclear sequences and floral characters, when using both AICc (ΔAICc = 486.5) and BIC scores (ΔBIC = 2788.1).

We found no evidence of correlated molecular and morphological rates when we analyzed the data using independent sister-pairs contrasts (*r*_*s*_* *= –0.002, *P* = 0.505; Fig. 8b), correlations of Bayesian mean posterior branch rates (*r*_*s*_ = 0.0835, *P* = 0.109; Fig. 8c), or correlations of Bayesian median posterior branch rates (*r*_*s*_ = 0.0122, *P* = 0.429; Fig. 8c). The BF gave very strong support to unlinking the clock models between nuclear and floral characters, with an average log BF of 41.1. Our Bayesian approaches failed to detect a correlation regardless of whether fossil calibrations were included or not (see Supplementary Material for the results of analyses without fossil calibrations).

## Discussion

We have shown through a comprehensive simulation study that correlated rates of evolution between molecular sequences and morphological characters can be detected under a variety of circumstances. The best-performing method was correlations of Bayesian branch rates, followed by root-to-tip distances, Bayesian model selection, independent sister-pairs contrasts, and lastly likelihood-based model selection. However, when taking computational burden into account, testing for correlations using root-to-tip distances is the most efficient method. Overall, methods had more power when the data had a moderate or high degree of among-lineage rate variation, and when at least 45 taxa or 100 morphological characters were sampled. When we applied these methods to an angiosperm data set, we found limited evidence for coupled evolutionary rates when analyzing nuclear DNA and floral characters. The estimation of root-to-tip distances might have been misled by missing data for floral traits. However, missing data are often unavoidable in morphological data sets, due to characters that are not common across species, difficulties in accessing suitable samples (Scholtz 2010; Wanninger 2015), and incompletely preserved fossil specimens. Although our simulations used evolutionary parameters that were empirically informed, they still represented an idealized form of the evolutionary process and yielded complete data sets. Below we discuss the results and implications of the simulation study before returning to the case study of angiosperms.

### Detecting Correlations Between Rates of Molecular and Morphological Evolution

Our simulation study has provided detailed evaluations of 5 methods of testing for correlations between rates of molecular and morphological evolution. We found that the 3 methods that used inferences from maximum-likelihood phylogenetic analysis required at least 100 morphological characters for accurate detection of rate correlations. Correlations of root-to-tip distances performed well, with a low rate of false positives. While statistical analyses of root-to-tip distances are hindered by the nonindependence of the data points (Rambaut et al. 2016), appropriate *P*-values can be computed using a permutation test (Garren 2017; Higgins 2004). Analysis using independent sister-pairs contrasts was able to detect rate correlations less frequently than the other methods that we evaluated, and this is likely to be due to the reduced number of data points that are sampled. For instance, for the tree including 111 species, root-to-tip distance correlations are based on 111 data points, whereas independent sister-pairs contrasts use only 35 data points.

When we used likelihood-based model selection to compare models with proportionate (linked) versus unlinked branch lengths, we consistently found support for the proportionate model even for data that had been generated by simulation with uncorrelated rates. This was probably because the proportionate model captures a substantial amount of variation while bringing only a modest increase in the number of parameters (Duchêne et al. 2020). The proportionate model was favored under almost all simulation settings, except when there was a large number of morphological characters. Of the 2 information criteria that were employed, model selection using the AICc yielded fewer false positives, since it penalizes model size less harshly than the BIC (Duchêne et al. 2020). However, we note that the AICc and BIC might not be appropriate for phylogenetic analyses under certain settings, particularly when using partition and mixture models (Susko and Roger 2020; Liu et al. 2023). For instance, AICc grows more biased when parameter space expands, and performs poorly in the presence of very short branches (Susko and Roger 2020).

The 2 Bayesian methods of testing for rate correlations showed highly contrasting performance in our simulation study. We found that correlations could be detected in our analyses of Bayesian branch rates even under less informative settings, such as when there were only 10 morphological characters. This strong performance was unexpected, given the typically large uncertainty in estimates of branch rates (e.g., Ho et al. 2005; Drummond et al. 2006), but can perhaps be attributed to the large number of data points sampled by the test (1 comparison per branch). Furthermore, removal of the shortest branches led to increased detection of rate correlations, particularly for simulation scenarios with less informative settings (Supplementary Material), but at the cost of a slightly increased rate of false positives. Compared with the likelihood-based approach, Bayesian model selection performed well when detecting correlated evolutionary rates, except when there was low among-lineage rate variation. It may be useful to compare the performance of other methods of marginal-likelihood estimation, such as path sampling or nested sampling (Skilling 2006; Russel et al. 2019).

Further evaluations of methods that test for correlations between molecular and morphological rates of evolution will be valuable, given that the dynamics of morphological evolution and the relationship to molecular evolution remain poorly understood (Lee and Palci 2015). Our simulations can be expanded to include a broader diversity of evolutionary rates, to reflect the estimates that have been obtained from empirical data. For example, rates of 0.01–0.5 changes/Myr have been inferred from morphological characters in hymenopteran insects, pufferfishes, and mammals (Klopfstein et al. 2019). Incorporating missing data in the simulations would increase the realism of the data sets and allow evaluation of the impacts of missing data on detecting correlations in evolutionary rates. We have not considered processes “external” to coding in DNA sequences, such as phenotypic plasticity and epigenetics, but these may shape adaptation and phenotypic changes over time (West-Eberhard 1989; Nylin and Wahlberg 2008). Our simulations did not test the influence of mismatched tree topology, which would likely hinder the detection of correlated rates. Furthermore, our simulations involved rates of morphological and molecular evolution that were either completely independent or perfectly correlated. We would expect the state of reality to lie somewhere between these 2 extremes, so that the degree of rate variability required to allow detection of correlated rates is likely to be even higher than suggested by our simulation study.

The performance of methods is likely to be worse in analyses of real data sets than of synthetic data sets because of the complexities of the evolutionary process in reality, and because of the shortcomings of our evolutionary models. This was demonstrated by our analyses involving mismatched clock models. Analyses implementing mismatched clock models led to a greatly inflated false-positive rate when among-lineage rate variation was low. This was likely to be due to the properties of the exponential relaxed clock, whereby the distribution of branch rates has a standard deviation equal to the mean (i.e., the coefficient of rate variation equals 1.0). The large dispersion of branch rates assumed under this model presents a substantial mismatch when data have been generated with low among-lineage rate variation. Analyses of these data using the exponential relaxed clock led to inaccurate inference of branch rates for the molecular and morphological trees, causing methods to fail when detecting rate correlations. While this result demonstrates the negative impact of model mismatch when there is low rate variation, the effects are likely to be less important in real data sets where among-lineage rate variation is much more substantial. Furthermore, the lack of false negatives when there is model mismatch indicates that the methods are generally capable of identifying the genuine absence of rate correlations.

### Studying the Dynamics of Morphological Rate Variation

The existence of a strict morphological clock has so far been rejected (Beck and Lee 2014; Lee and Palci 2015; O’Reilly et al. 2015; Lee 2016; Tarasov 2019). This is reinforced by the apparent lack of a “common mechanism” governing the evolution of morphological characters, with the pattern of among-character rate variation differing across branches (Goloboff et al. 2019). However, by testing for correlations between molecular and morphological evolutionary rates, we can better understand the dynamics of the morphological clock. While a broad link between molecular and morphological change is expected (Simpson 1953), the rate of phenotypic mutation has been observed to be orders of magnitude larger than that of genotypic mutations in eukaryotic organisms (Bürger et al. 2006). The higher rate of phenotypic mutations might be caused by a lack of selective pressure to reduce the rate of mutation below a certain threshold, effectively rendering phenotypic mutation rates independent of genotypic mutation rates (Bürger et al. 2006). Furthermore, a decoupling of morphological and molecular evolutionary rates would be consistent with stronger selection acting on phenotypic traits compared with molecular sequences (Lande 1976; Machado 2020; Simões and Pierce 2021; Machado et al. 2022). Recent empirical studies have demonstrated this decoupling in various groups of vertebrate animals (Bromham et al. 2002; Lee et al. 2013; Halliday et al. 2019; Simões et al. 2020b).

Estimating morphological rates of evolution is fraught with uncertainty, and the distribution of rates across taxa and over time is largely undescribed (Simpson 1944; Schopf 1984). Unlike molecular data, the collection of morphological data is “infinitely extensible”; there is no upper boundary on the total number of characters and states that can be considered (Oyston et al. 2022) because there are no objectively defined categories such as the 20 amino acids or 4 nucleotides found in molecular data (Dávalos et al. 2014; Lee and Palci 2015; Barba-Montoya et al. 2021). The morphological characters that are selected for phylogenetic inference are usually chosen for their diagnostic utility, so invariant and rapidly evolving characters are typically excluded (Lewis 2001; Wright and Hillis 2014). However, there is growing acknowledgement of the importance of autapomorphies for evolutionary analysis, and they are increasingly being included in morphological data sets (King et al. 2017; Paterson et al. 2019; Simões et al. 2020a, 2022a; King 2021).

Previous work has shown that Bayesian and maximum-parsimony phylogenetic analyses of morphological data have greater accuracy for data that have been generated under stochastic processes rather than being subject to selection (Keating et al. 2020). This indicates that at a macroevolutionary scale, the dynamics of morphological evolution might deviate from the Mk model, which is a simplified version of the general multiple-rate asymmetrical Mk model, originally introduced for morphological data (Pagel 1994; Goloboff et al. 2019; Keating et al. 2020). Although the inadequacy of the Mk model is often assumed to hamper phylogenetic inference using morphological characters, it might not be a substantial problem unless homoplasy is particularly extensive (Jablonski and Finarelli 2009) or when rates are extremely high (Reyes et al. 2018). Apart from these circumstances, Bayesian inference using the Mk model seems to be relatively robust and can accurately infer topologies and branch lengths under a broad range of conditions (Wright and Hillis 2014). In any case, model adequacy can be evaluated using methods such as cross-validation or posterior predictive simulations (Brown 2014; Duchêne et al. 2016; Brown and Thomson 2018).

### Evolutionary Rates in Angiosperms

Our analysis of data from 111 angiosperms failed to detect a correlation between the evolutionary rates of nuclear DNA and floral traits. The 30-character floral data set that we analyzed might not have been sufficiently informative, so our results will require confirmation using a larger data set. Additionally, the floral data set that we examined excluded hypervariable characters, such as floral color. The resulting characters in the floral data set were all slowly evolving, at rates below 0.006 changes/Myr. These low rates were suited to the primary goal of ancestral state reconstruction for which this data set was assembled (Sauquet et al. 2017).

The molecular data set used to test for correlations between rates of floral and sequence evolution included 410 protein-coding, single-copy nuclear genes, obtained by sequencing the vegetative tissue transcriptomes of plant species (One Thousand Plant Transcriptomes Initiative (2019)). These 410 protein-coding genes likely control phenotypic expression across a broad range of characters. However, the set of 30 curated floral traits only represents a small proportion of the total phenotypic traits of each flowering plant species. Assessing a larger number of morphological traits will not only lend more power to the analyses but will also provide a more accurate reflection of the overall rate of morphological trait evolution. Such a data set is not yet available for flowering plants across a broad phylogenetic sample because of the considerable work required in its assembly. Studies could possibly be done with resources such as the global “TRY” plant database (Kattge et al. 2020), which is composed mostly of vegetative traits. Generally, floral traits are under intense sexual selection (Barrett 2010), which might influence the detection of correlated rates. Indeed, Barraclough and Savolainen (2001) found a very strong correlation between the evolution of molecular sequences and floral traits, but a weak correlation when analyzing vegetative traits.

Overall, the result might correctly reflect a more general uncoupling of molecular and morphological rates in angiosperms. Assuming that molecular evolution is largely gradual, a decoupling of evolutionary rates between the nuclear genome and floral characters suggests a departure from a model of gradual morphological change, that is, that morphological evolution is not proportional to time (Halliday et al. 2019; Parins-Fukuchi et al. 2021; Stull et al. 2021; Pagel et al. 2022). Pulses of morphological change have occurred throughout plant evolution, possibly at speciation events (Eldredge and Gould 1972), with notable episodes corresponding to the introduction of vascular plants in the Devonian and the diversification of angiosperms in the Late Cretaceous (Leslie et al. 2021). However, testing whether pulses of “punctuated” evolution occur at speciation events is not possible due to the design of this study. All nucleotide substitutions and morphological character changes were simulated as occurring gradually along branches. Further tests of methods for detecting rate correlations can be performed using simulations that involve more complex modes of evolutionary change (e.g., Manceau et al. 2020; Janzen et al. 2022).

A lack of an association between rates of floral character and molecular evolution would also be consistent with floral evolution being driven by changes at specific loci (Kimura 1968; Barrier et al. 2001; Davies and Savolainen 2006; Duret 2008; Gaut et al. 2011). The mutations that produce phenotypic change might occur largely in adaptive and regulatory genes, while many genomic mutations are neutral in their impact on fitness (Kimura 1968, 1983). Indeed, a large proportion of the morphological diversity amongst flowering plants can be attributed to specialized interactions between angiosperms and their insect pollinators (Darwin 1862; Friis et al. 2006; Cappellari et al. 2013; Benton et al. 2021; Asar et al. 2022). Furthermore, in our study, we were limited to examining evolutionary change in protein-coding genes, which only included the first 2 sites of each codon. Testing for correlations separately using rates of nonsynonymous and synonymous substitution will allow further insights into the relative importance of selection and drift (Barrier et al. 2001).

## Concluding Remarks

We have shown that correlations between molecular and morphological evolutionary rates can be detected under the conditions explored in our simulation study. However, the complexities of how morphological evolution proceeds, and whether this is effectively described by current evolutionary models and approaches, will ultimately determine whether the rates of morphological character evolution and their correlates can be accurately reconstructed in practice. While we did not find evidence of correlated evolutionary rates between angiosperm genomic DNA and floral characters, the question of whether the rates of genotypic and phenotypic evolution are correlated in angiosperms should be addressed with a larger morphological data set.

Our study has implications for combined analyses of molecular and morphological data, where branch lengths between data sets are often linked as a default approach (Nylander et al. 2004; O’Reilly et al. 2015). The results of our simulation study lead us to suggest that future studies should use morphological character matrices of at least 100 characters; this would allow for partitioning of the morphological data set, which has been demonstrated to improve the precision of divergence date estimates and accuracy of branch-length estimates (Lee 2016; Caldas and Schrago 2019; Neumann et al. 2021). Furthermore, partitioning of morphological data can also provide independent estimates of evolutionary rates across subdivisions of the phenotype, as recently done for empirical data sets using relaxed clocks (Paterson et al. 2019; Simões and Pierce 2021). This work should not only be extended to larger data sets, but should also span across the Tree of Life, to help elucidate the processes that drive macroevolutionary change. Finally, the methods examined in the present study are not restricted to analyses of molecular and morphological evolution but can also be used to test for correlations in rates between symbionts and their hosts or between organellar and nuclear genomes in plants.

## Supplementary Material

Data available from the Dryad Digital Repository: https://doi.org/10.5061/dryad.7wm37pw01

## Data Availability

Data sets and scripts to perform simulations and empirical analyses are available on dryad via: https://doi.org/10.5061/dryad.7wm37pw01. The One Thousand Plant Transcriptomes Initiative (2019) sequence alignment used in empirical analyses is available in Zenodo via: https://doi.org/10.5281/zenodo.3255100.
